# Guidelines for the Li–Fraumeni and heritable *TP53*-related cancer syndromes

**DOI:** 10.1038/s41431-020-0638-4

**Published:** 2020-05-26

**Authors:** Thierry Frebourg, Svetlana Bajalica Lagercrantz, Carla Oliveira, Rita Magenheim, D. Gareth Evans, The European Reference Network GENTURIS, Nicoline Hoogerbrugge, Marjolijn Ligtenberg, Marleen Kets, Rianne Oostenbrink, Rolf Sijmons, Gareth Evans, Emma Woodward, Marc Tischkowitz, Eamonn Maher, Rosalie E. Ferner, Stefan Aretz, Isabel Spier, Verena Steinke-Lange, Elke Holinski-Feder, Evelin Schröck, Thierry Frebourg, Claude Houdayer, Chrystelle Colas, Pierre Wolkenstein, Vincent Bours, Eric Legius, Bruce Poppe, Kathleen Claes, Robin de Putter, Ignacio Blanco Guillermo, Gabriel Capella, Joan Brunet Vidal, Conxi Lázaro, Judith Balmaña, Hector Salvador Hernandez, Carla Oliveira, Manuel Teixeira, Svetlana Bajalica-Lagercrantz, Emma Tham, Jan Lubinski, Karolina Ertmanska, Bela Melegh, Mateja Krajc, Ana Blatnik, Sirkku Peltonen, Marja Hietala

**Affiliations:** 1 Department of Genetics, Rouen University Hospital and Inserm U1245, Normandie University, UNIROUEN, Normandy Centre for Genomic and Personalized Medicine, Rouen, France; 2 Hereditary Cancer Unit, Department of Clinical Genetics, Karolinska University Hospital, Stockholm, Sweden; 3 i3S—Instituto de Investigação e Inovação em Saúde & Institute of Molecular Pathology and Immunology of the University of Porto, and Porto Comprehensive Cancer Center, Porto, Portugal; 4 Community Representative, Berlin, Germany; 5 Manchester Centre for Genomic Medicine, Division of Evolution and Genomic Sciences, University of Manchester, MAHSC, St Mary’s Hospital, Manchester University Hospitals NHS Foundation Trust, Manchester, UK; 6 Radboud University Medical Center, Nijmegen, The Netherlands; 7 NKI Antoni van Leeuwenhoek hospital, Amsterdam, The Netherlands; 8 Erasmus Medical Center, Rotterdam, The Netherlands; 9 University Medical Center, Groningen, The Netherlands; 10 Genomic Medicine, Central Manchester Foundation Trust, Manchester, UK; 11 Cambridge University Hospitals NHS Foundation Trust, Cambridge, UK; 12 Guy’s and St. Thomas’ NHS Foundation Trust, London, UK; 13 University Hospital Bonn, Bonn, Germany; 14 Medizinisch Genetisches Zentrum, Munich, Germany; 15 Hereditary Cancer Syndrome Center Dresden, Dresden, Germany; 16 Rouen University Hospital, Rouen, France; 17 Institut Curie, Paris, France; 18 University Hospital Henri Mondor-National Referral Center, Créteil, France; 19 University Hospital, Liege, Belgium; 20 University Hospital Leuven, Leuven, Belgium; 21 Ghent University Hospital, Ghent, Belgium; 22 Hospital Universitari Germans Trias i Pujol y ICO Badalona, lnstitut Catala d’Oncologia, Barcelona, Spain; 23 Hospital Sant Joan de Déu, Barcelona, Spain; 24 Porto Comprehensive Cancer Center, Porto, Portugal; 25 Karolinska University Hospital, Stockholm, Sweden; 26 Pomeranian Medical University - University Clinical Hospital n1, Szczecin, Poland; 27 University of Pécs, Pécs, Hungary; 28 Institute of Oncology, Ljubljana, Slovenia; 29 Turku University Hospital, Turku, Finland

**Keywords:** Cancer screening, Risk factors

## Abstract

Fifty years after the recognition of the Li–Fraumeni syndrome (LFS), our perception of cancers related to germline alterations of *TP53* has drastically changed: (i) germline *TP53* alterations are often identified among children with cancers, in particular soft-tissue sarcomas, adrenocortical carcinomas, central nervous system tumours, or among adult females with early breast cancers, without familial history. This justifies the expansion of the LFS concept to a wider cancer predisposition syndrome designated heritable *TP53-*related cancer (h*TP53*rc) syndrome; (ii) the interpretation of germline *TP53* variants remains challenging and should integrate epidemiological, phenotypical, bioinformatics prediction, and functional data; (iii) the penetrance of germline disease-causing *TP53* variants is variable, depending both on the type of variant (dominant-negative variants being associated with a higher cancer risk) and on modifying factors; (iv) whole-body MRI (WBMRI) allows early detection of tumours in variant carriers and (v) in cancer patients with germline disease-causing *TP53* variants, radiotherapy, and conventional genotoxic chemotherapy contribute to the development of subsequent primary tumours. It is critical to perform *TP53* testing before the initiation of treatment in order to avoid in carriers, if possible, radiotherapy and genotoxic chemotherapies. In children, the recommendations are to perform clinical examination and abdominal ultrasound every 6 months, annual WBMRI and brain MRI from the first year of life, if the *TP53* variant is known to be associated with childhood cancers. In adults, the surveillance should include every year clinical examination, WBMRI, breast MRI in females from 20 until 65 years and brain MRI until 50 years.

## Introduction

### From Li–Fraumeni syndrome to heritable *TP53*-related cancers

Heterozygous germline *TP53* alterations were identified in 1990 in the Li–Fraumeni syndrome (LFS), described in 1969 by Frederick Li and Joseph Fraumeni [1–3]. The p53 protein normally acts as a guardian of the genome, and if DNA damage occurs, p53 triggers a response based on transcription regulation of numerous genes involved in cell cycle, DNA repair, apoptosis, senescence and cellular metabolism. LFS is characterised by a strong familial aggregation of cancers, early-onset of tumours and wide tumour spectrum, including the so-called core LFS cancers: soft-tissue sarcomas (STS), osteosarcomas, adrenocortical carcinomas (ACC), central nervous system (CNS) tumours and very early-onset female breast cancers, occurring before 31 years. Fifty years after the initial clinical recognition of the syndrome, germline alterations of *TP53* are mainly identified among children with cancers or among adult females with breast cancers, in both cases often without familial history of cancer. For this reason, our perception of cancers related to germline alterations of *TP53* has drastically changed through time [4–6]. The diversity of clinical presentations associated with germline *TP53* alterations justifies the expansion of the LFS concept to a wider cancer predisposition syndrome designated heritable *TP53-*related cancer (h*TP53*rc) syndrome. Criteria for germline *TP53* variant screening named ‘Chompret criteria’ have been sequentially updated ([6]; see below recommendation 1). Regardless of familial history, the detection rate of disease causing germline *TP53* variants has been estimated to be: 50–80% in children presenting with ACC [6–9] or choroid plexus carcinomas [6, 10]; up to 73% in children with rhabdomyosarcoma of embryonal anaplastic subtype [11] and between 3.8 and 7.7% in females with breast carcinoma before 31 years of age [12]. These data demonstrate that familial history of cancer should not be mandatory when considering genetic testing of *TP53*. The frequency of presentations without familial cancer history is explained both by the contribution of de novo variants to h*TP53*rc syndrome, which has been estimated to be between 7 and 20% [13, 14], and the incomplete penetrance of germline *TP53* variants. Beside the Chompret criteria, recent reports and experience of certain centres justify to extend *TP53* testing to other clinical presentations suggestive of a germline *TP53* alteration: Children and adolescents with hypodiploid acute lymphoblastic leukaemia [15, 16], otherwise unexplained sonic hedgehog-driven medulloblastoma [17], jaw osteosarcoma (see below recommendation 2) and patients who develop a second primary tumour within the radiotherapy field of a first core *TP53* tumour which occurred before 46 years (see below recommendation 3).

### Interpretation of germline *TP53* variants

Because the *TP53* gene is currently included in several cancer gene panels broadly used in genetic testing, the number of *TP53* tests performed in non-suggestive clinical situations has exponentially increased. Whereas the interpretation of *TP53* variants predicted to result in loss of function, such as nonsense or frameshift deletions or insertions, is usually obvious, the interpretation of missense variants, representing the majority, is often challenging and requires specific expertise. The most common consequence of germline variants causing h*TP53*rc syndrome is the functional inactivation of the protein. Classification of *TP53* missense variants, in agreement with the ACMG/AMP guidelines, is based on several considerations including *phenotypical data* (identified in patients fulfilling the Chompret criteria); *frequency of the variant in the general population*, as reported by the Genome Aggregation Database (https://gnomad.broadinstitute.org/), *bioinformatics predictions* of the variant impact on protein or RNA splicing using different algorithms, and *functional analyses* of the variants performed using different *in vitro* assays performed either in yeast or cultured cells [18–21] (http://p53.iarc.fr/). Optimised and stringent ACMG/AMP criteria for a specific classification of germline *TP53* variants, integrating the above considerations, are being developed by a *TP53* variant curation expert panel, under the umbrella of ClinGen. This will allow a progressive allocation or re-classification of *TP53* variants into the different ACMG/AMP classes. Since the distinction between class 5 (pathogenic) and class 4 (likely pathogenic) variants is particularly subtle for *TP53* variants, these variants are designated in the current ERN guideline as ‘disease-causing’ variants.

### The question of mosaicism

The presence of mosaic *TP53* alterations should be considered in patients with sporadic cancers strongly suggestive of a disease-causing *TP53* variant, such as childhood ACC, choroid plexus carcinoma, and breast cancer before 31 years of age and in patients with multiple primary tumours belonging to the *TP53* core tumour spectrum [14]. The absence of detectable *TP53* variants after analysis of blood DNA using NGS, even performed at a high depth, does not guarantee the absence of mosaic alterations which can be restricted to other tissues than blood. Therefore, a complete screening for *TP53* disease causing variants in highly suggestive situations should include analysis of tumour tissue, which is so far not systematically performed. In contrast, the detection of a *TP53* variant in a small fraction of NGS reads from blood DNA does not always correspond to a mosaic alteration [22–25] and molecular geneticists should be aware of two pitfalls: the first corresponds to circulating tumour DNA, commonly observed in patients with metastatic cancers. For instance, the detection of a *TP53* variant in the blood from a patient with metastatic high grade serous ovary carcinoma is likely to correspond to circulating tumour DNA, considering the very high frequency of somatic *TP53* alterations in these malignancies (>95%); the second is due to clonal haematopoiesis, corresponding to the occurrence, in hematopoietic stem cells of somatic *TP53* alterations conferring a growth advantage. Clonal haematopoiesis was initially reported in patients over 70 years of age, but can be detected from 30 years of age. The frequency of clonal haematopoiesis is increasing with age, tobacco use and exposure to chemotherapy or radiotherapy [22–25]. Therefore, when a *TP53* variant is detected in a small fraction of NGS reads from blood, it is critical, before concluding that the presence represents a mosaic *TP53* alteration, to respect the following rules: (i) consider the clinical presentation (suggestive or not of the presence of a disease-causing *TP53* variant) and medical history (treatments, metastases, etc…) and (ii) confirm the presence of the variant in the tissue from which the tumour originated. Further confirmation in an unaffected tissue with no lymphocyte content, such as a hair follicle, skin biopsy or nail clippings, should also be considered if circulating tumour DNA is suspected from metastatic disease.

### Cancer risk associated with germline *TP53* variants

A challenge when dealing with *TP53* variant carriers is to estimate the cancer risk or penetrance associated with each specific *TP53* variant, and this cancer risk has recently been revisited. Indeed, the cumulative cancer incidence of germline disease-causing *TP53* variants was initially calculated using information mainly from familial cases and was estimated to 73–100% by age 70, with risks close to 100% in women [26–28]. The predominance of familial cases likely results in an ascertainment bias and an overestimation of disease penetrance. This should be regarded in perspective with the prevalence in the general population of germline disease-causing *TP53* variants, which was recently estimated, based on a conservative approach, to be in the magnitude of 1 among 4500 individuals [29]. In childhood, the main tumour risks are ACC, STS, osteosarcomas and CNS tumours whereas the main tumour risk in adults corresponds to female breast cancers, female *TP53* variant carriers have an excessively high risk of developing breast cancer before 31. There is no known elevated risk of male breast cancer [4–6, 26–28, 30]. There is a perception that colorectal cancer is associated with germline pathogenic *TP53* variants [31–33]. However, the corresponding studies suffer from methodological limitations and interpretation of some reported *TP53* variants is problematic. Families with a germline *TP53* variant and an additional history of colorectal cancer in the pedigree may have increased risk of colorectal cancer. This increased risk is, however, not associated with the *TP53* variant itself and, on the basis of the published studies, a high risk of colorectal cancer can be confidently excluded in carriers of disease-causing *TP53* variants.

The penetrance of germline disease-causing *TP53* variants is variable. One factor explaining the variability of this penetrance is the type of the variant itself: some of the p53 proteins bearing missense mutations are classified as dominant-negative due to their ability to complex and reduce the transcriptional activity of wild-type p53 protein, producing malfunctioning or non-functioning p53 tetramers. These dominant-negative missense *TP53* variants are usually detected in families with childhood cancers and are generally highly penetrant. In contrast, null variants (frameshift or nonsense variants, splicing variants, large genomic rearrangements, and non-dominant-negative missense variants), are predominantly identified in families with mostly adult cancers and have a lower disease penetrance [6]. A remarkable example of a low penetrant, but still disease-causing variant, is the non-dominant-negative missense p.Arg337His variant, present in 0.3% of the population from Southern Brazil and associated with a founder effect [34–36]. The difference in the clinical severity between dominant-negative missense variants and the remaining ones is explained by a difference in their biological impact on the p53 transcriptional activity. Indeed, measurement of the transcriptional response to DNA damage in cells harbouring heterozygous *TP53* variants, has shown that dominant-negative missense variants have a more drastic impact on p53 DNA binding and transcriptional response to DNA damage, than the other types of heterozygous alterations [19]. The clinical annotation of the variants and updated functional data should allow progressively, dichotomising disease-causing *TP53* variants in ‘high cancer risk’ and ‘low cancer risk’ alleles.

The phenotypic variability observed within the same family (e.g. a child affected with cancer and the parent, carrier of the same variant, being not affected in childhood) strongly supports the existence of genetic modifying factors and their identification represents, at the present time, a top priority in the field. It is more and more evident that phenotypic expression in carriers of *TP53* disease-causing variants is also dependent on environmental factors, as germline *TP53* variants may turn p53 into a protein permissive to oncogenic stress.

### The impact of radio and chemotherapy on the development of subsequent primary tumours

Germline *TP53* variant carriers have a remarkably high incidence of subsequent primary tumours, which may occur in more than 40% of *TP53* variant carriers [6, 27]. Subsequent primary tumours often develop after the exposure of *TP53* variant carriers to radio and/or chemotherapy treatments. The demonstration of the contribution of radiotherapy and conventional chemotherapy to the development of subsequent primary tumours in these carriers came from consistent observations of sequential development of multiple tumours after treatment by chemo- or radiotherapy of a first tumour and the development of tumours within the radiotherapy field [6]. A cause-effect was strongly supported by studies of the impact of chemo- and radiotherapy in lymphocytes with wild-type or mutant *TP53* genotype and LFS mouse models [37]. Therefore, in cancer patients, testing for disease-causing *TP53* variants must absolutely take place before starting treatment and if a disease-causing *TP53* variant is found, priority should be given to surgical or ablative treatments, avoiding radiotherapy when possible and using preferably non-genotoxic chemotherapies.

### Surveillance protocols

Surveillance protocols for carriers bearing disease-causing *TP53* variants have recently been elaborated in the framework of an international consortium coordinated by Canadian and US teams [38, 39]. These protocols indicate that carriers should undergo abdominal ultrasound every 3–4 months, annual whole-body MRI (WBMRI) and annual brain MRI (the first with gadolinium enhancement) from the first year of life. Additionally, female carriers should undergo annual breast MRI from the age of 20 years onwards. The option of risk-reducing mastectomy may be discussed on a case-by-case basis [39]. Several international studies, mostly performed without gadolinium-based contrast agents (GBCAs), have confirmed the efficiency of WBMRI, with an overall estimated detection rate of 7% for new and localised primary cancers on a first prevalent screen [40–46]. Given that GBCAs may be retained for months or years in several organs, multiple GBCAs administrations should probably be avoided in germline *TP53* variant carriers and only macrocyclic GBCAs, which are apparently less retained in the body [47], should be used.

### Scope of the guidelines

These guidelines have been elaborated by members of the European Reference Network (ERN) on the Genetic Tumour Risk Syndromes (GENTURIS) in order to integrate the available information with clinical utility and to assist health care professionals in the identification and management of patients with germline disease-causing *TP53* variants, causing heritable *TP53-*related cancer (h*TP53*rc) syndrome. In particular, these guidelines take into account the diversity of clinical presentations associated with germline *TP53* variants, the variability of the *TP53* variant penetrance, the role of radiotherapy and chemotherapy in the development of subsequent primary tumour and the medical benefit of surveillance protocols. These guidelines do not signify nor intend to be a legal standard of care, they should support clinical decision making, but never replace clinical professionals. This is particularly critical for h*TP53*rc syndrome, considering the diversity of clinical expression related to germline *TP53* variants.

### Methods followed

The ERN GENTURIS h*TP53*rc syndrome guideline was established by molecular and clinical geneticists and clinicians with expertise in paediatrics, oncology, or radiology, as well as affected individuals and parent representatives. The Guideline Development Group was supported by a Core Writing Group composed of ERN GENTURIS HCP members from different member states and who are recognised experts and specialised in molecular oncobiology and/or clinical practice and/or in the diagnosis and management of h*TP53*rc. The Core Writing Group leads had joint meetings with a Patient Advisory Group composed of affected individuals and parent representatives that have experience with h*TP53*rc syndrome. The elaboration of these guidelines has then actively involved external experts from different speciality areas that are relevant to the scope of the guideline.

The guidelines were elaborated on the basis of 337 published articles extracted from Pubmed, using the following terms: (screening [title/abstract] OR surveillance [title/abstract] OR detection [title/abstract]) AND (LFS[title] OR Li–Fraumeni [Title] OR TP53 [title]) AND ‘humans’ [MeSH Terms]. Additional articles were requested from experts in the field and references of all the articles were considered. As is typical for many rare diseases, the volume of peer-reviewed evidence available to consider for these guidelines was small and came from a limited number of articles, which typically reported on small samples or series. To balance the weight of both published evidence and quantify the wealth of expert experience and knowledge, we have used for evidence grading the following scale: (i) *strong evidence*: consistent evidence and new evidence unlikely to change recommendation and expert consensus; (ii) *moderate evidence*: expert consensus or majority decision but with inconsistent evidence or significant new evidence expected and (iii) *weak evidence*: inconsistent evidence AND expert agreement.

## Recommendations

### Cancer patients who should be tested for germline disease-causing *TP53*^a^

**Table:** 

Recommendation 1	All patients who meet the modified ‘Chompret Criteria’ should be tested for germline *TP53* variants: • *Familial presentation*: proband with a *TP53* core tumour (breast cancer, soft-tissue sarcoma, osteosarcoma, central nervous system tumour, adrenocortical carcinoma) before 46 years AND at least one first- or second-degree relative with a core tumour before 56 years; *or* • *Multiple primitive tumours*: proband with multiple tumours, including 2 *TP53* core tumours, the first of which occurred before 46 years, irrespective of family history; *or* • *Rare tumours*: patient with adrenocortical carcinoma, choroid plexus carcinoma, or rhabdomyosarcoma of embryonal anaplastic subtype, irrespective of family history; *or* • *Very early-onset breast cancer*: Breast cancer before 31 years, irrespective of family history
Recommendation 2	Children and adolescents should be tested for germline *TP53* variants if presenting with: • Hypodiploid acute lymphoblastic leukaemia (ALL); *or* • Otherwise unexplained *sonic hedgehog*-driven medulloblastoma; *or* • Jaw osteosarcoma
Recommendation 3	Patients who develop a second primary tumour, within the radiotherapy field of a first core *TP53* tumour which occurred before 46 years, should be tested for germline *TP53* variants
Recommendation 4	a. Patients older than 46 years presenting with breast cancer without personal or familial history fulfilling the ‘Chompret Criteria’ should not be tested for germline *TP53* variants
	b. Any patient presenting with isolated breast cancer and not fulfilling the ‘Chompret Criteria’, in whom a disease-causing *TP53* variant has been identified, should be referred to an expert multi-disciplinary team for discussion
Recommendation 5	Children with any cancer from southern and south-eastern Brazilian families should be tested for the p.R337H Brazilian founder germline *TP53* variant

^a^Testing for disease-causing *TP53* variants should be performed before starting treatment in order to avoid in variant carriers, if possible, radiotherapy and genotoxic chemotherapy and to prioritise surgical treatments.

### Pre-symptomatic testing recommendations

**Table:** 

Recommendation 6	Adult first-degree relatives of individuals with germline disease-causing *TP53* variants should be offered testing for the same germline *TP53* variant
Recommendation 7	The testing in childhood, from birth, of first-degree relatives of individuals with germline disease-causing *TP53* variants should be systematically offered, if updated knowledge, based on databases and registries, shows that the variant can be considered as a high cancer risk *TP53* variant conferring a high cancer risk in childhood: • The index case has developed a childhood cancer; *or* • Childhood cancers have been observed within the family; *or* • This variant has already been detected in other families with childhood cancers; *or* • This variant corresponds to a dominant-negative missense variant
Recommendation 8	The testing in childhood of first-degree relatives of individuals with germline disease-causing *TP53* variants should *not* be systematically offered, if updated knowledge, based on databases and registries, shows that the variant can be considered as a low cancer risk *TP53* variant and does not confer a high cancer risk in childhood: • The index case has not developed a childhood cancer; *and* • Childhood cancers have not been observed within the family; *and* • This variant has not already been reported in other families with childhood cancers; *and* • This variant does not correspond to a dominant-negative missense variant
Recommendation 9	The testing in childhood of first-degree relatives of individuals with germline disease-causing *TP53* variants should be discussed with their parents if cancers have occurred in early adulthood (before the age of 31 years) within the family, *or* if there is insufficient evidence in the databases or registries to determine the childhood cancer risk. • This discussion should address the burden, and uncertain benefits, of surveillance in childhood, before a decision is made whether to test the child for germline disease-causing *TP53* variants.

### Surveillance recommendations in carriers of germline disease-causing *TP53* variants

**Table:** 

Recommendation 10	In children, clinical examination should be performed every 6 months, with specific attention to signs of virilization or early puberty, and measurement of blood pressure
	In adults, clinical examination should be performed annually with specific attention, in patients who received radiotherapy, to occurrence of basal cell carcinomas within the radiotherapy field
Recommendation 11	In adults, WBMRI without gadolinium enhancement should be conducted annually
Recommendation 12	In individuals with high cancer risk *TP53* variants or previously treated by chemotherapy or radiotherapy, WBMRI without gadolinium enhancement, should be conducted annually, from birth
Recommendation 13	In female individuals, breast MRI should be conducted annually, from 20 years until 65 years
Recommendation 14	In children, from birth, and in adolescents (<18 years), abdominal ultrasound for the detection of adrenocortical carcinoma should be conducted at least every 6 months
Recommendation 15	In children, from birth, and in adolescents (<18 years), when abdominal ultrasound does not allow a proper imaging of the adrenal glands, measurement of urine steroids, for detection of ACC, should probably be conducted at least every 6 months
Recommendation 16	In adults until 50 years, brain MRI should be conducted annually
Recommendation 17	In individuals with high cancer risk *TP53* variants, brain MRI should be conducted from birth, annually
Recommendation 18	If surveillance includes brain MRI, at least the first (prevalence) scan should be conducted using dedicated brain MRI with Gadolinium enhancement
Recommendation 19	In children, if surveillance includes annual brain MRI, this should alternate with the WBMRI, so that the brain is imaged at least every 6 months
Recommendation 20	Colonoscopy should be performed, from 18 years, every 5 years, only if the carrier received abdominal radiotherapy for the treatment of a previous cancer, *or* if there is a familial history of colorectal tumours suggestive of an increased genetic risk

### Guideline summary: surveillance protocol in carriers of GERMLINE disease-causing *TP53* variants

**Table:** 

Exam	Periodicity	Age to start	Age to end	Condition	Evidence^a^
Clinical examination with, in children, specific attention to signs of virilisation or early puberty and measurement of blood pressure and, in patients who received radiotherapy, to occurrence of basal cell carcinomas within the radiotherapy field	Every 6 months	Birth	17 years		Moderate
	Annual	18 years	–		Moderate
Whole-Body MRI without gadolinium enhancement	Annual	Birth	–	High cancer risk *TP53* variant^b^ or patient previously treated by chemotherapy or radiotherapy	Moderate
		18 years	–		Strong
Breast MRI	Annual	20 years	Until 65 years		Strong
Brain MRI^c^	Annual	Birth	18 years	High cancer risk *TP53*variant	Moderate
		18 years	Until 50 years		Moderate
Abdominal ultrasound	Every 6 months	Birth	Until 18 years		Strong
Urine steroids	Every 6 months	Birth	Until 18 years	When abdominal ultrasound does not allow a proper imaging of the adrenal glands	Weak
Colonoscopy	Every 5 years	18 years	–	Only if the carrier received abdominal radiotherapy for the treatment of a previous cancer or if there is a familial history of colorectal tumours suggestive of an increased genetic risk	Weak

^a^This grading is based on published articles and expert consensus.

^b^A germline disease-causing *TP53* variant should be considered as ‘high risk’ if the index case has developed a childhood cancer; *or* childhood cancers have been observed within the family; *or* this variant has already been detected in other families with childhood cancers; *or* this variant corresponds to a dominant-negative missense variant.

^c^The first scan should be conducted with I.V. Gadolinium enhancement; in children, brain MRI should alternate with the Whole-Body MRI, so that the brain is imaged at least every 6 months.
